# Correlation Between Hyperlipidemia-Related Diseases and Thorax/Thigh Circumference Ratio Along with Body Condition Score in Dogs Focusing on Molecular Mechanism: A Pilot Study and Literature Review

**DOI:** 10.3390/life14111441

**Published:** 2024-11-07

**Authors:** Kyuhyung Choi

**Affiliations:** 1 Department of Veterinary Pathology, College of Veterinary Medicine, Seoul National University, Seoul 08826, Republic of Korea; cokonut@naver.com or kyudac@snu.ac.kr; 2 Bundang New York Animal Hospital, Seongnam 13637, Republic of Korea

**Keywords:** thorax/thigh circumference ratio, body condition score, hyperlipidemia, obesity, patellar luxation, ApoA-1, high-density lipoprotein

## Abstract

There are some limitations to using the body condition score (BCS) for client education to prevent obesity, hyperlipidemia-related diseases, and orthopedic diseases in dogs because it is hard to quantify in detail. Especially in small dogs, patellar luxation is a common orthopedic disease that is related to obesity and the hind leg muscle. In this pilot study, the author evaluated the thorax/thigh circumference ratio as a prognostic evaluation index, along with the BCS, for assessing patellar dislocation and other hyperlipidemia-related diseases and states such as hypertriglyceridemia and obesity-related orthopedic disease in small dogs. Eleven client-owned dogs were selected randomly among patients that visited Bundang New York Animal Hospital, South Korea from June 2021 to August 2024. According to the results, triglycerides (TG) showed a negative correlation with thorax/thigh value (R = −0.585, *p*-value = 0.059) and a strong positive correlation with thigh circumference (R = 0.749, *p*-value = 0.008). Total cholesterol (TC) showed a strong positive correlation with thigh circumference (R = 0.776, *p*-value = 0.005), whereas the thorax/thigh value showed a negative correlation with the medial patella luxation (MPL) grade with low significance (R = −0.343, *p*-value = 0.302). These data indicate that thigh circumference can be an excellent negative indicator for hyperlipidemia and thorax/thigh value shows no correlation with medial patella luxation, which has many factors such as varus and trochlear groove. Despite the limitations of this study due to the small sample size, this pilot study is significant as it is the first trial to introduce a new indicator for monitoring hyperlipidemia at home by using a simple tape measure. Also, the author reviews molecular pathways including the ApoA-1, ApoE, and LPL genes, which are related to hyperlipidemia, to explain the results.

## 1. Introduction

The body condition score was first introduced in dairy cows to evaluate their productivity [1]. It is traditionally used in the form of a five-grade or nine-grade scale, and a higher grade reflects more body fat. A score of three points on the five-grade system or five points on the nine-grade system is suitable for the healthy maintenance of cows. The BCS is also applied to other animals, including small animals such as feline and canine species. The criteria of the BCS are visibility of the rib, lumber vertebrae, and pelvic bone and discernible waist in dogs, and an ideal state of body is four to five points on the nine-grade system [2,3]. As this is a somewhat subjective element, evaluation of the BCS can vary slightly depending on the person measuring it [4], and the scores are not subdivided in detail, so it is quite difficult to monitor subtle differences [5]. Failure in monitoring subtle differences using the BCS can lead to obesity [6], hyperlipidemia [7,8], and other endocrine diseases such as Cushing’s disease. Hyperlipidemia commonly results in diabetes, hypothyroidism, and Cushing’s syndrome in dogs [9] as well as humans [10,11]. To date, the correlation between hyperlipidemia and orthopedic diseases has not been clearly documented in dogs, and there are only a few studies on the effects of hyperlipidemia on knee tendons in rats [12,13]. However, the anatomy of dogs and rats is very different, so the results may not be applicable to dogs [14,15]. In contrast, there are many studies on the correlation between obesity, BCS, and orthopedic diseases, and numerous studies have attempted to develop monitoring indicators and prognostic evaluation biomarkers to assist in assessing the BCS [16,17,18,19]. In humans, abdominal fat is closely related to hypertension and the waist/hip ratio, which is positively correlated with cardiovascular disease in epidemiological research [20]. Additionally, high-density lipoprotein (HDL), which is known as ‘good cholesterol’ that cleans fat in vessel to lower the possibility of cardiovascular disease, is reported to be negatively correlated to truncal fat and waist/hip ratio [21]. Moreover, abdominal fat can increase insulin resistance, leading to a higher risk of diabetes mellitus [22]. Therefore, in order to manage abdominal fat effectively, various indicators such as waist/hip ratio and waist/chest ratio, along with Body Mass Index (BMI), have been developed [23]. Additionally, although BMI-related abdominal fat is commonly associated with diabetes mellitus, hypertension, and cardiovascular diseases, hind leg muscle circumference has been shown to be a better indicator than BMI in respiratory diseases such as Chronic Obstructive Pulmonary Disease (COPD) [24]. In contrast, there are few biomarkers or indicators other than the BCS for monitoring obesity and related diseases even though their importance is increasing in veterinary medicine [25,26,27]. Moreover, in veterinary medicine, there are breed differences even in the same species, so the same BCS does not always reflect the same body fat, especially in dogs [25]. For example, Schnauzers are genetically prone to hyperlipidemia because Very-Low-Density Lipoprotein (VLDL) and chylomicrons accumulate abnormally to a large degree in this breed compared to other breeds [28]. Thus, Schnauzers may be more susceptible than other breeds to hyperlipidemia-related diseases such as pancreatitis, and it has been demonstrated that it may be related to mutation of the SPINK1 gene [29]. Therefore, the author would like to introduce a new indicator, thorax/thigh ratio in dogs, which resembles the waist/hip ratio in humans. Comprehensive monitoring of body fat in a detailed but simple way is key to managing health successfully in veterinary medicine as well as in human medicine. The significance and originality of this study lies in its development of an indicator that can be used alongside the BCS and body weight to diagnose hyperlipidemia and related diseases.

## 2. Materials and Methods

**Figure 1:**
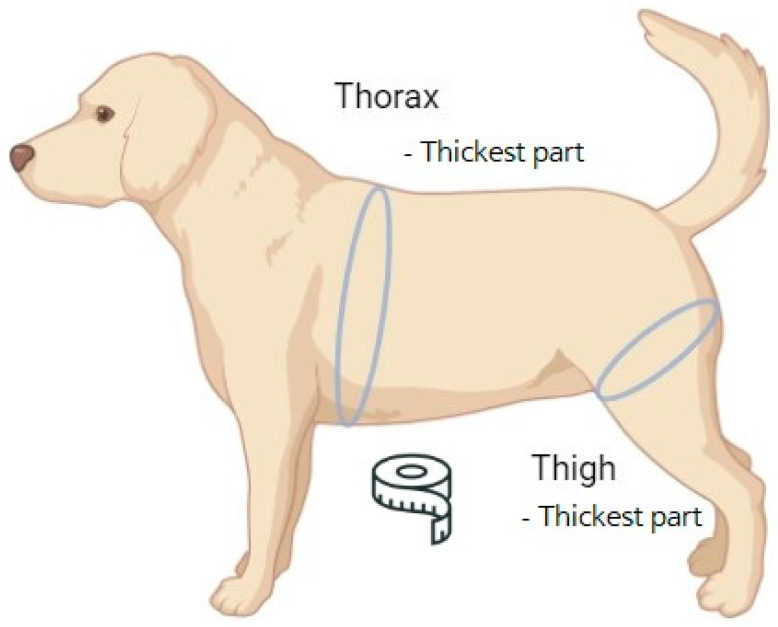
How to measure the thorax/thigh ratio.

Blood samples were collected from eleven client-owned dogs visiting Bundang New York Animal Hospital, a general practice located in South Korea. The dogs were selected randomly among patient aged between 1 to 17 years old from different breeds and sexes who visited hospital from June 2021 to August 2024 (Table 1). All blood samples were collected from the cephalic vein of the dogs after physical examination (measuring body weight, BCS, thorax, thigh circumference, patella luxation, gait) following 6 h of fasting [30] without anesthesia or sedation and centrifuged at 14,500 RPM for 1 min, and the sera were immediately and directly analyzed using a DRI-CHEM NX500 (Fujifilm, Tokyo, Japan), a dry chemistry analyzer. The samples were measured directly without dilution at room temperature. The circumference of the thigh and thorax [31] of the dogs was measured manually using a tape measure (Figure 1). JASP (version, 0.19.1, the JASP team, Amsterdam, The Netherlands) was used for data analysis (Pearson correlation, independent sample *t*-test) and graph creation (https://jasp-stats.org, accessed on 20 August 2024). Amino acid sequence analysis was performed using Vectorbuilder (Version 2.1.92, VectorBuilder Inc., Chicago, IL, USA, https://en.vectorbuilder.com/tool/sequence-alignment.html, accessed on 20 August 2024).

## 3. Results

As expected, the BCS and thorax circumference showed a positive correlation (R = 0.487, *p*-value = 0.128), and the BCS and TC showed a significant strong positive correlation (R = 0.683, *p*-value = 0.021). The BCS and TG also showed a significant strong positive correlation, as expected (R = 0.587, *p*-value = 0.057). The BCS has been used as an indicator of obesity, and these data confirm that the BCS is indeed a valid standard.

Unfortunately, the thorax/thigh value and medial patella luxation showed a negative correlation with low significance (R = −0.343, *p*-value = 0.302) since patella luxation is multifactorial. The thorax/thigh value and TC showed a negative correlation (R = −0.461, *p*-value = 0.154). The thorax/thigh value and TG showed a negative correlation with low significance (R = −0.316, *p*-value = 0.344). The TG and thorax/thigh value showed a significant negative correlation (R = −0.585, *p*-value = 0.059). These data indicate that the thorax/thigh value has limited application at present but it shows potential, so further investigation involving a larger sample is needed.

Interestingly, total cholesterol and thigh (left side) circumference showed a positive correlation (R = 0.561, *p*-value = 0.073). TC and thigh (right side) circumference showed a significant strong positive correlation (R = 0.776, *p*-value = 0.005). Triglyceride and thigh (right side) circumference also showed a significant strong positive correlation (R = 0.749, *p*-value = 0.008). Triglyceride and thigh (left side) circumference also showed a significant strong positive correlation (R = 0.711, *p*-value = 0.014) which is consistent in both sides rather than total cholesterol (For detailed information, see the Supplementary Materials). As there are only a few studies that focused on the correlation between hyperlipidemia and thigh circumference in veterinary and human medicine, further investigation using a large sample would be beneficial to assess the utilization of thigh circumference as a prognostic indicator for hyperlipidemia.

## 4. Discussion

It has been revealed that although the BCS involves subjective elements, it is a reliable standard for diagnosing obesity, which is positively correlated with hyperlipidemia in dogs. To more easily monitor obesity and related diseases, the thorax/thigh circumference ratio was first introduced in this study. Although the thorax/thigh value is insignificantly correlated with medial patella luxation, as it is complex disease [32], the thorax/thigh value has a strong negative correlation with triglycerides. It can be explained that small thigh circumference may lead to an increasing level of triglycerides in humans [33], so it can be applicable to dogs, but further investigation of mechanism is needed. In fact, patellar dislocation commonly results from hindleg angular limb deformity and trochlear groove depth [34]. Therefore, the thorax/thigh ratio can be a useful indicator for hypertriglyceridemia even though there are clear limitations when applied to orthopedic diseases such as patellar dislocation and hindleg limping. Additionally, as there are many studies on the correlation between hyperlipidemia and thigh circumference in humans, validating this correlation in the veterinary field, especially in small animals, can help monitor animal health at home more effectively.

**Figure 2:**
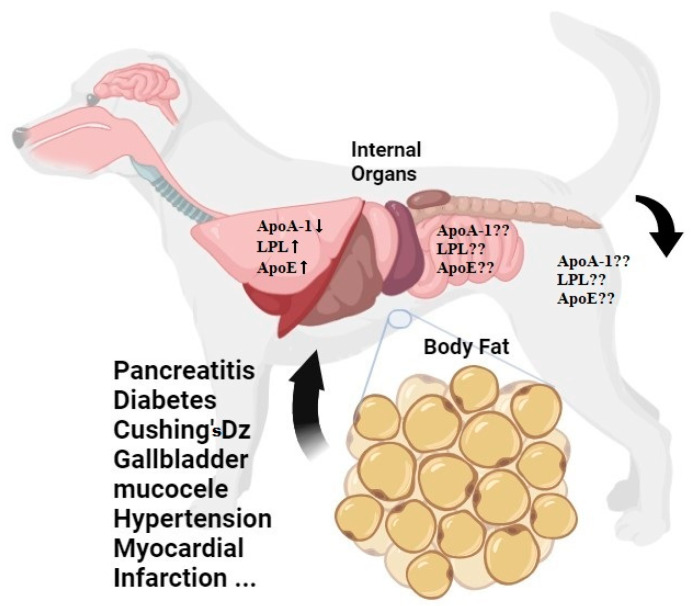
Schematic illustration of gene expression.

However, hyperlipidemia may be related to underlying diseases such as gallbladder sludge (Table 1, case 4, Pomeranian) or genetic predisposition (for example, idiopathic familial hyperlipidemia in Schnauzers) rather than abdominal fat or body weight in dogs, so it is important to check the underlying disease and breed difference beforehand. In fact, in cases 4 and 6, hyperlipidemia was observed in dogs with larger thighs than other dogs of similar weight (Table 1). Hindleg lipid deposition changes with aging and may be related to metabolic changes [35], implying that a large portion of the hindleg of cases 4 and 6 may consist of lipids. In addition to hindleg fat, abdominal fat is closely related to diabetes, hyperlipidemia, high blood pressure, and cardiovascular disease in humans, and there have been recent attempts to monitor abdominal fat by quantifying it with Computed Tomography (CT). The importance of abdominal fat in diseases has recently emerged in the veterinary field (Figure 2), and there are some studies that evaluated abdominal fat using CT in dogs recently [36,37,38,39].

The results of this study are significant because hyperlipidemia and obesity can be easily monitored by simply measuring body and leg circumferences, along with the BCS and body weight, at a local hospital or at home. Although this study has limitations due to the small number of dogs, the thorax/thigh ratio and thigh circumference, along with the BCS, can be secondary indicators for hyperlipidemia, which can be used to improve obesity monitoring systems to provide proper diet and supplements to prevent or treat related diseases.

## 5. Reviewing Molecular Mechanism Focus on Related Genes

**Figure 3:**
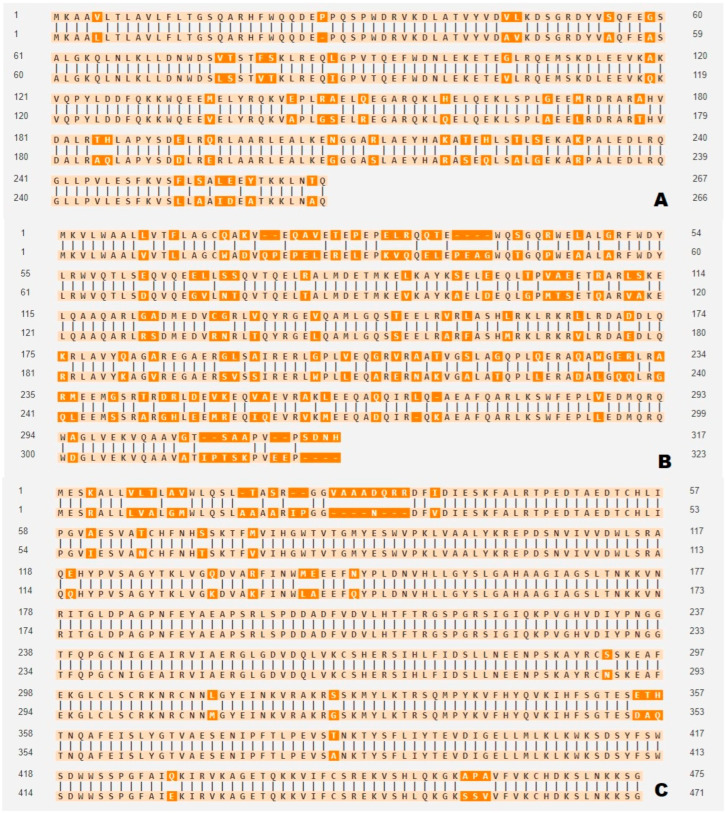
(**A**): Amino acid alignment of Apoa1 (Sequence 1 length:267 Sequence 2 length:266 Alignment length: 267 Identity: 227/267, 85.02%, Similarity: 246/267, 92.13%, Gaps: 1/267, 0.37%), (**B**): ApoE (Sequence 1 length:317 Sequence 2 length:323 Alignment length: 328 Identity: 226/328, 68.90%, Similarity: 268/328, 81.71%, Gaps: 16/328, 4.88%), (**C**): LPL (Sequence 1 length:475 Sequence 2 length:471 Alignment length: 478, Identity: 437/478, 91.42%, Similarity: 455/478, 95.19%, Gaps: 10/478,2.09%, sequence 1 human, sequence 2 dog).

From the aspect of the molecular level, ApoA-1, ApoE, and lipoprotein lipase (LPL) genes are related to hyperlipidemia [40]. In particular, ApoA-1, which is known to encode a major component of HDL, may be an explanation for thigh circumference and total cholesterol correlation, because waist/hip ratio is strongly negatively associated with HDL and positive correlated with total cholesterol [41] since thigh circumference resembles the waist/hip ratio in human according to this preliminary data. There are physiological differences between humans and dogs, and thigh fat storage is strongly correlated with body weight and abdominal fat in dogs [42], which could be an interesting explanation for this pilot study’s results. Also, there is only 68.9% of identity and 81.7% of similarity between the human and dog ApoE amino acid sequences (Figure 3B), which is relatively lower than human and dog ApoA1 (Figure 3A) and human and dog LPL (Figure 3C). Additionally, the ApoE gene which is known to positively correlated to cardiovascular disease and hyperlipidemia is cell-specifically expressed and mainly expressed in the liver and other organs including the small intestine and brain in humans and mice [43] (Figure 2). There is no documented estimation of ApoE mRNA concentrations in hindleg or thigh tissue in dogs; a follow up study will elucidate the fat distribution and, putting these together, it will provide clues to a deeper understanding of metabolic diseases from a comparative molecular point of view in humans and dogs. For example, we can compare human and dog ApoE mRNA concentrations in thigh fat, and investigate their comparative correlation with diabetes mellitus.

**Figure 4:**
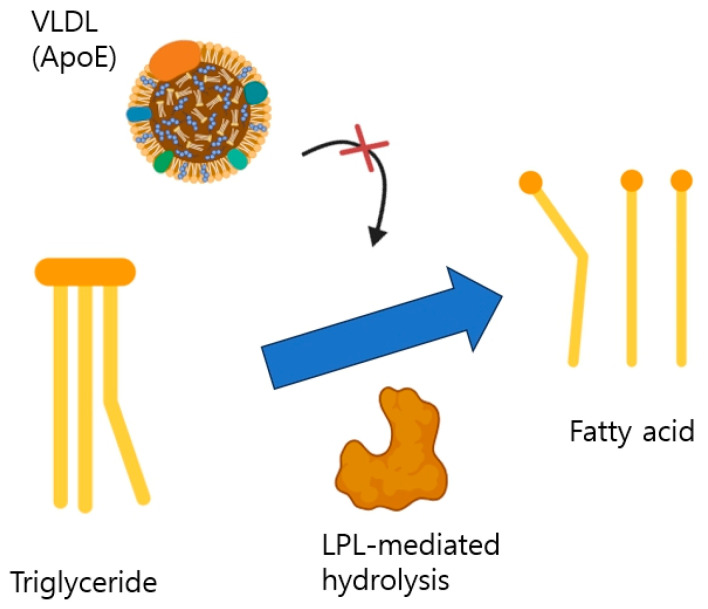
Illustration of how ApoE and LPL affect lipid metabolism.

Moreover, LPL activity is known to be positively correlated with hindleg exercise in dogs [44]. LPL is highly expressed in coronary artery disease patients [40] and a variant of LPL is closely related to hyperlipidemia [45]. Also, ApoE limits LPL-mediated hydrolysis of triglycerides [46] (Figure 4). Therefore, monitoring hindleg circumference at home after exercise has a plausible capacity to reflect hyperlipidemia blood profile.

Genetic mutation and polymorphism on ApoA-1, ApoE, and LPL is also closely related to hyperlipidemia [47] according to previous research by Baroni, 2003. Although this research focused on cardiovascular disease, the authors described that mutation of those genes may disturb the metabolic pathway of lipids and lead to hyperlipoproteinemia. Even after that, there are many documents which have analyzed correlation between hyperlipidemia and mutation of apolipoprotein until now, 2024 [48,49,50]. To elucidate the molecular pathway of hyperlipidemia in dogs, which includes many factors such as various associated genes and polymorphism and tissue-specific expression, the author recommends the single-cell RNA profiling technique which is generally used in human metabolic disease these days [51], since there is no article published where the technique is applied to hyperlipidemia dogs. This may reveal the genetic pathway of apolipoproteins organ-specifically as in the schematic illustrations shown in Figure 2 and Figure 4.

## 6. Conclusions

The author briefly reviewed the molecular pathway of hyperlipidemia in dogs through pilot study data. The data showed that triglycerides showed a strong positive correlation with thigh circumference in both sides consistently (left, R = 0.711, *p*-value = 0.014, right, R = 0.749, *p*-value = 0.008) and total cholesterol in only one side (right, R = 0.776, *p*-value = 0.005), even though thorax/thigh circumference ratio failed to be a new indicator comparable to the waist/hip circumference ratio in humans. The ApoE gene may be crucial for storage of fat in the thigh since it limits LPL-mediated hydrolysis of triglycerides and LPL activity is positively correlated to thigh exercise which can raise metabolic rate and help with metabolic disease. A follow up study utilizing the single-cell RNA technique would elucidate the hyperlipidemia molecular pathway of dogs by comparing human and dog ApoE mRNA concentrations at the tissue level individually.

## Figures and Tables

**Table 1:** Signalment and concurrent disease of 11 dogs used in correlation and comparison study. (y: year, m: month, BCS: body condition score 1 to 9, All blood work units: mg/dL, LP: left-sided pain, RP: right-sided pain, N: normal).

#	Breed	Age	Sex	Body Weight	BCS	Thigh Circumference (L/R), cm	Thorax Circumference, cm	Thorax/Thigh Ratio	Total Cholesterol (111~312)	Triglycerides (30~133)	Patella Luxation Grade (L/R)	Gait	Concurrent Disease
1	Pug	17 y	CM	7.95 kg	6/9	19/19	45	2.37/2.37	229	104	2/2	N	Patella luxation
2	Pomeranian	3 y 6 m	CM	3.75 kg	5.5/9	13/16	36	2.76/2.25	287	101	0/0	N	None
3	Bichon Frise	1 y 3 m	SF	2.73 kg	4/9	15/12	28	1.86/2.33	120	57	1.5/2	N	Patella luxation
4	Pomeranian	6 y 6 m	CM	3.4 kg	6/9	19/19	25	1.3/1.3	500	432	2/1	N	Gallbladder sludge
5	Mix	1 y	SF	2.95 kg	4/9	14/14	26	1.86/1.86	162	45	2/1	LP	Patella luxation
6	Pomeranian	5 y	SF	3.9 kg	7.5/9	18/22	43	2.38/1.95	500	333	3.5/2.5	LP	Obesity
7	Pomeranian	3 y	SF	2.5 kg	6/9	15.5/16	22	1.46/1.375	293	111	3/2.5	N	Patella luxation
8	Pomeranian	3 y 4 m	SF	4.85 kg	5.5/9	14/14	30	2.14/2.14	338	43	2.5/2.5	N	Patella luxation
9	Bichon Frise	2 y 7 m	SF	3.6 kg	6/9	14/13	29	2.07/2.23	120	87	2/1.5	N	Patella luxation
10	Maltese	6 y 6 m	CM	5.35 kg	6/9	16/17	43	2.68/2.52	305	95	2/1	N	Patella luxation
11	Maltese	5 y 2 m	CM	5 kg	5.5/9	15.5/14.5	32.5	2.09/2.24	317	86	2.5/2.5	RP	Patella luxation

## Data Availability

Data supporting this study are included within the article and/or Supporting Materials.

## Supplementary Materials

The following supporting information can be downloaded at: https://www.mdpi.com/article/10.3390/life14111441/s1, Supplementary Datasets S1.

## Abbreviations

BCS	Body Condition Score
TC	Total Cholesterol
TG	Triglycerides
MPL	Medial Patellar Luxation
HDL	High-Density Lipoprotein
