# Supplementary material for: Correlation Between Hyperlipidemia-Related Diseases and Thorax/Thigh Circumference Ratio Along with Body Condition Score in Dogs Focusing on Molecular Mechanism: A Pilot Study and Literature Review
Source: Life (Basel). 2024 Nov 7;14(11):1441. doi: 10.3390/life14111441 (PMC11595692; doi:10.3390/life14111441)
Supplement: Supplementary file 1 [file life-14-01441-s001.zip › life-3277807-supplementary.pdf]

Dataset S1

Pearson's Correlations

Variable		BCS	Thorax circumference
1. BCS	Pearson's r	—	
	p-value	—	
2. Thorax circumference	Pearson's r	0.487	—
	p-value	0.128	—

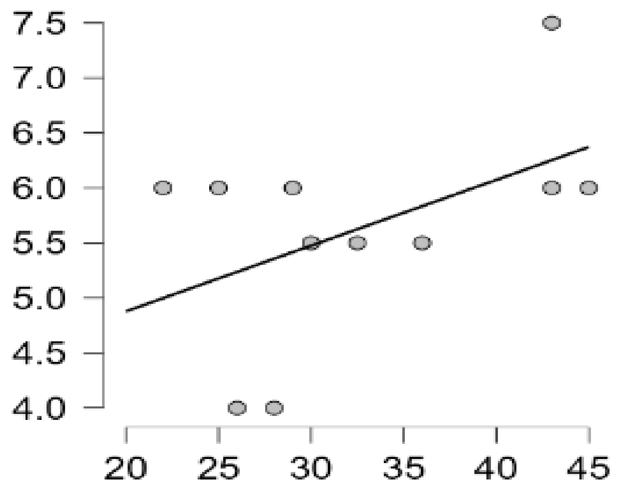

BCS and thorax circumference showed a positive correlation (R = 0.487, p-value = 0.128).

Pearson's Correlations

Variable		BCS	TC
1. BCS	Pearson's r	—	
	p-value	—	
2. TC	Pearson's r	0.683	—
	p-value	0.021	—

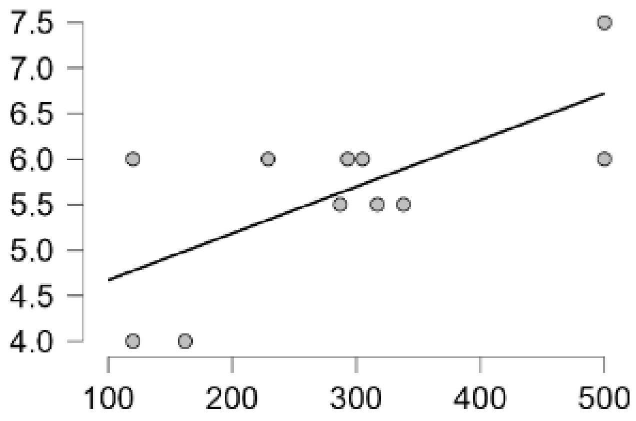

BCS and TC showed a significant strong positive correlation, as expected (R = 0.683, p-value = 0.021).

Pearson's Correlations ▼

Variable		BCS	TG
1. BCS	Pearson's r	—	
	p-value	—	
2. TG	Pearson's r	0.587	—
	p-value	0.057	—

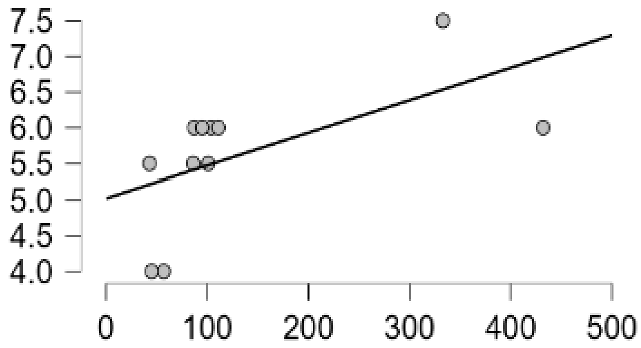

BCS and TG also showed a significant strong positive correlation, as expected (R = 0.587, p-value = 0.057).

Pearson's Correlations ▼

Variable		Thorax/Thigh (L)	MPL (L)
1. Thorax/Thigh (L)	Pearson's r	—	
	p-value	—	
2. MPL (L)	Pearson's r	−0.343	—
	p-value	0.302	—

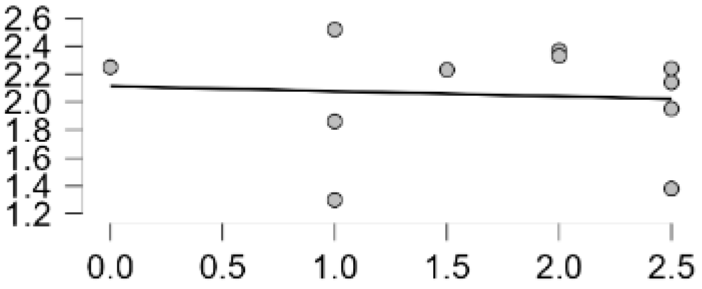

Thorax/thigh value and MPL showed a negative correlation with low significance (R = -0.343, p-value = 0.302).

Pearson's Correlations ▼

Variable		Thorax/Thigh (R)	TC
1. Thorax/Thigh (R)	Pearson's r	—	
	p-value	—	
2. TC	Pearson's r	−0.461	—
	p-value	0.154	—

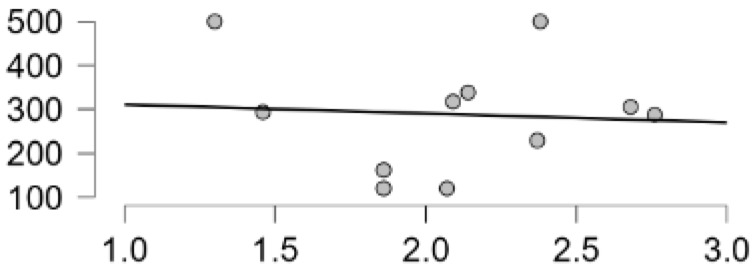

Thorax/thigh value and TC showed a negative correlation (R = -0.461, p-value = 0.154).

Pearson's Correlations ▼

Variable		Thorax/Thigh (L)	TG
1. Thorax/Thigh (L)	Pearson's r	—	
	p-value	—	
2. TG	Pearson's r	−0.316	—
	p-value	0.344	—

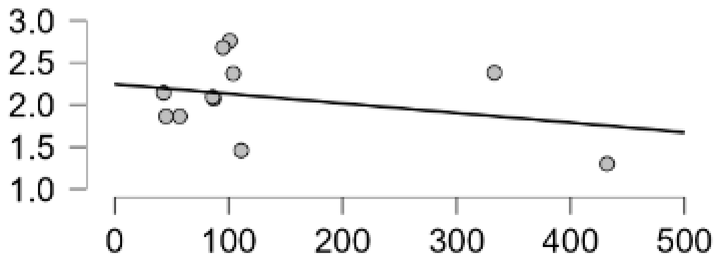

Thorax/thigh value and TG showed a negative correlation with low significance (R = -0.316, p-value = 0.344).

Pearson's Correlations ▼

Variable		TG	Thorax/Thigh (R)
1. TG	Pearson's r	—	
	p-value	—	
2. Thorax/Thigh (R)	Pearson's r	−0.585	—
	p-value	0.059	—

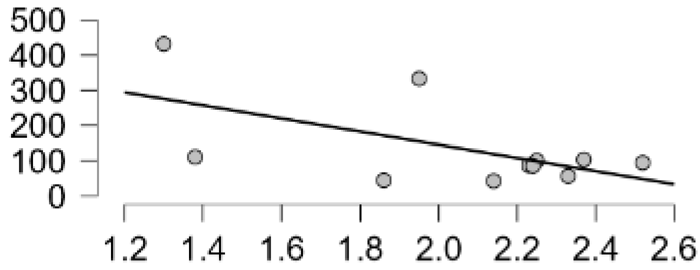

TG and thorax/thigh value showed a significant negative correlation (R = -0.585, p-value = 0.059).

Pearson's Correlations ▼

Variable		TC	Thigh (L)
1. TC	Pearson's r	—	
	p-value	—	
2. Thigh (L)	Pearson's r	0.561	—
	p-value	0.073	—

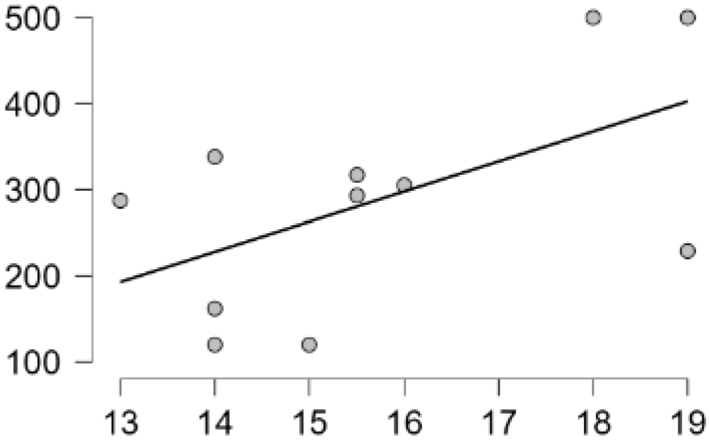

TC and thigh (left side) circumference showed a positive correlation (R = 0.561, p-value = 0.073).

Pearson's Correlations ▼

Variable		TC	Thigh (R)
1. TC	Pearson's r	—	
	p-value	—	
2. Thigh (R)	Pearson's r	0.776	—
	p-value	0.005	—

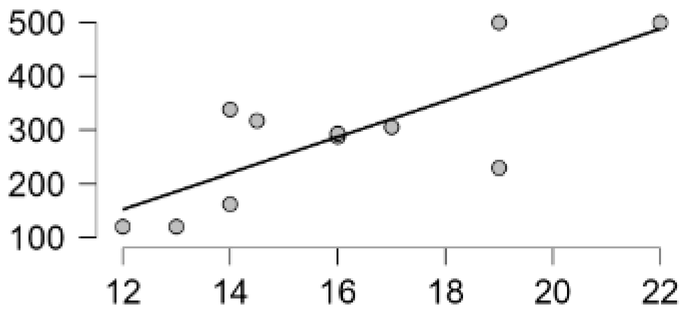

TC and thigh (right side) circumference showed a significant strong positive correlation (R = 0.776, p-value = 0.005).

Pearson's Correlations

Variable		Thigh (R)	TG
1. Thigh (R)	Pearson's r	—	
	p-value	—	
2. TG	Pearson's r	0.749	—
	p-value	0.008	—

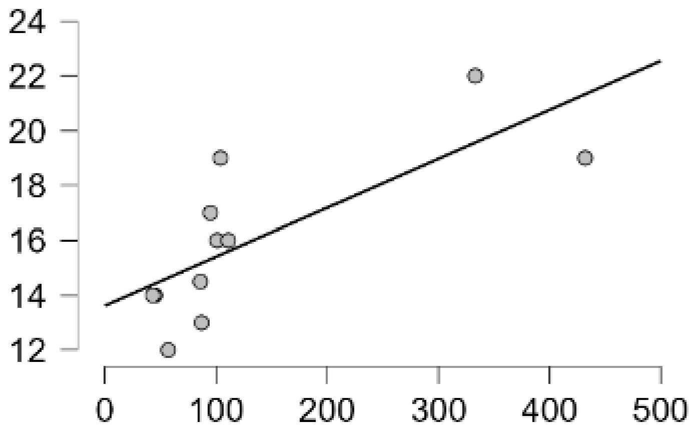

TG and thigh (right side) circumference showed a significant strong positive correlation (R = 0.749, p-value = 0.008).

Pearson's Correlations ▼

Variable		TG	Thigh (L)
1. TG	Pearson's r	—	
	p-value	—	
2. Thigh (L)	Pearson's r	0.711	—
	p-value	0.014	—

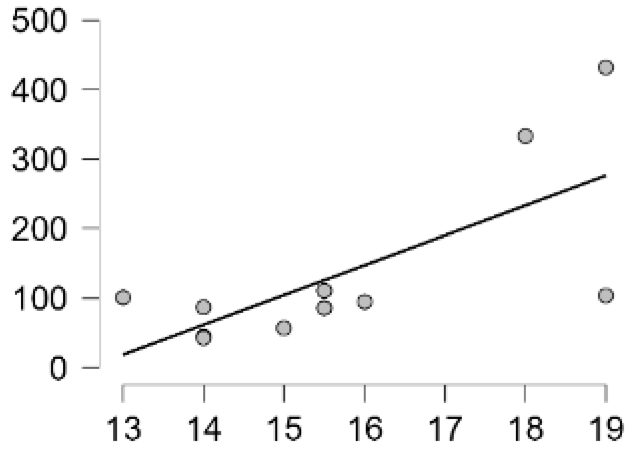

TG and thigh (left side) circumference showed a significant strong positive correlation (R = 0.711, p-value = 0.014).
